# COVID-19 vaccine effectiveness studies in Nigeria: Quo vadis?

**DOI:** 10.7189/jogh.12.03055

**Published:** 2022-08-03

**Authors:** Oluwatosin Wuraola Akande, Ehimario Uche Igumbor, Kelly Osezele Elimian, Cornelius Ehizokhai Ohonsi, Lilian Nwozor, Okanke Oden, Emmanuel Nsa Ekpenyong, Nnaemeka Ndodo, Ifeanyi F Ike, Magdalene Egede, William Nwachukwu, Amedu M Onoja, Jenson Gawain Fofah, Reuben Ishiaku Azi, Chinwe L Ochu, Ifedayo M Adetifa

**Affiliations:** 1 Nigeria Centre for Disease Control (NCDC), Abuja, Nigeria; 2 Nigeria COVID-19 Research Coalition (NCRC), Abuja, Nigeria; 3 School of Public Health, University of the Western Cape, Cape Town, South Africa; 4 Department of Global Public Health, Karolinska Institutet, Stockholm, Sweden; 5 Federal Ministry of Health, Abuja, Nigeria; 6 International Society for Infectious Diseases, Massachusetts, USA; 7 London School of Hygiene and Tropical Medicine, London, UK

The “lickety-split” development of COVID-19 vaccines 326 days from when the SARS-COV-2 virus was first sequenced is indeed one of the public health successes of the 21st century. Particularly because an 18-month target was initially considered reasonable, and having achieved this success, a “moonshot” goal to ensure that a vaccine is available within 100 days after the next pandemic pathogen is recognized has been set [1].

The vaccine efficacy outcome in clinical trials is evaluated as the level of protection conferred on individuals against the target disease under “controlled conditions”, while vaccine effectiveness (VE) assesses vaccine performance in “real-world settings”. Given the contrasting epidemiology of COVID-19 especially in terms of health outcomes globally, it is essential to systematically collect and analyse VE data to provide evidence to guide context-specific policies for the COVID-19 response, including the vaccination programme. This is particularly relevant in engendering trust in many African countries such as Nigeria that were not included in the initial trials that established the efficacy of the COVID-19 vaccine (**Figure 1**).

**Figure 1:**
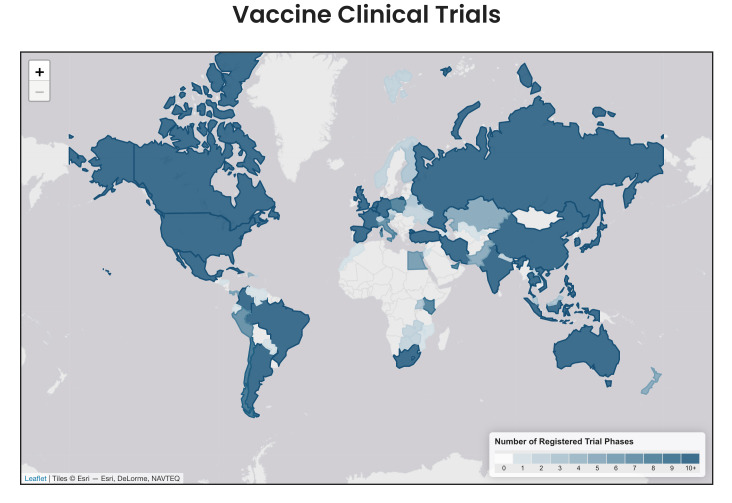
Map showing the distribution of COVID-19 vaccine trials conducted globally.

In this commentary, we highlight the importance of undertaking locally designed COVID-19 VE studies in Nigeria offer a review of current efforts to coordinate such studies, and summarize the initial operational considerations in implementing these studies in Nigeria.

## IMPLEMENTING THE COVID-19 VACCINE EFFECTIVENESS STUDY IN NIGERIA

As part of the national public health response to COVID-19, Nigeria began vaccine rollout in March 2021, about a year after the country reported its index case. Owing to the need for tailored evidence on VE in the country to guide policy and practice, the Nigeria Centre for Disease Control (NCDC) commenced plans to implement a study titled, “The Evaluation of COVID-19 Vaccine Effectiveness in Preventing Severe COVID-19 Disease among Adults in Nigeria” in February 2021. This NCDC-led study was designed as a multi-institutional collaboration with other governmental, non-governmental, and academic/research institutions, including the National Primary Health Care Development Agency, National Agency for Food and Drug Administration and Control, World Health Organisation (WHO), Joint United Nations Programme on HIV/AIDS, Africa Field Epidemiology Network, Nigeria COVID-19 Research Coalition (NCRC), and a network of tertiary health facilities managing COVID-19 cases as the study implementation sites.

**Figure:**
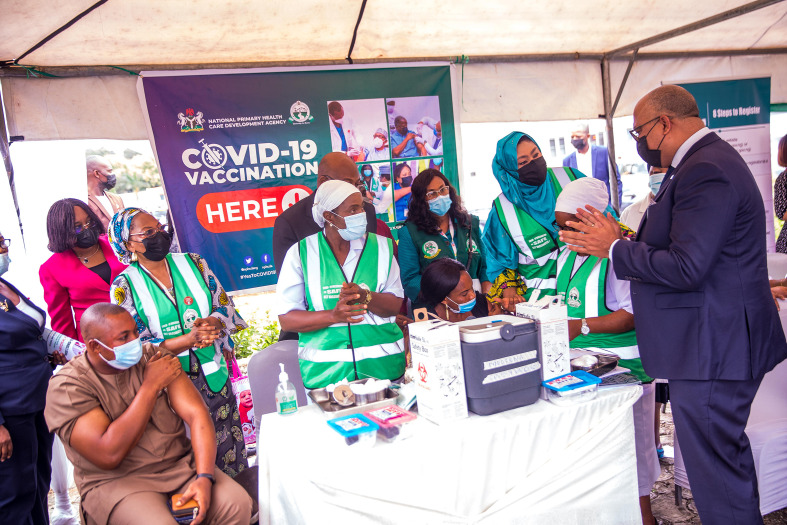
Photo: Driving COVID-19 vaccination in Nigeria - since March 2021, community outreach and vaccination campaigns have been key to the country's COVID-19 response. Source: www.IKPstudios.com

The primary aim of this study is to evaluate the post-introduction effectiveness of a complete schedule of SARS-CoV-2 vaccines among adults in Nigeria against severe COVID-19, using a test-negative case-control (TNCC) design. The study will estimate VE in preventing severe COVID-19 disease by vaccine type, number of vaccine doses received, identifiable target cohorts (subgroups of comorbidity, history of previous COVID-19 infection, etc.), time since vaccination, and viral strain. The TNCC study design is commonly used in estimating the VE of Influenza vaccines, and it is now being increasingly used in COVID-19 VE studies [2-6].

Adaptation of global study protocols for health research is a common practice in Africa because it allows for standardisation and facilitates comparison of results/outcomes [7]. The development of this study protocol was guided by the WHO’s guideline on the evaluation of COVID-19 VE and the minimum sample size (890 cases and 2670 controls) was estimated using O’Neill’s formula for calculating the protective efficacy of vaccines in case-control studies [8,9]. Ethical approval of the study was obtained from the National Health Research Ethics Committee and the institutional research ethics committees of the selected implementation sites.

Although COVID-19 VE evaluations can be assessed with a variety of study designs, the TNCC has been identified as the most efficient and least biased of the VE study designs for COVID-19 in a majority of settings [9]. This design utilises the results of the Polymerase Chain Reaction (PCR) tests for individuals with a high index of suspicion of COVID-19. The requirement of additional PCR testing beyond the routine health service provision is not required, thus saving on resources, and reducing the risk of disease transmission during sample collection among health workers. Furthermore, ethical dilemmas associated with other study designs around the use of placebo or following a group of unvaccinated individuals who are eligible for vaccination are curtailed.

**Figure 2:**
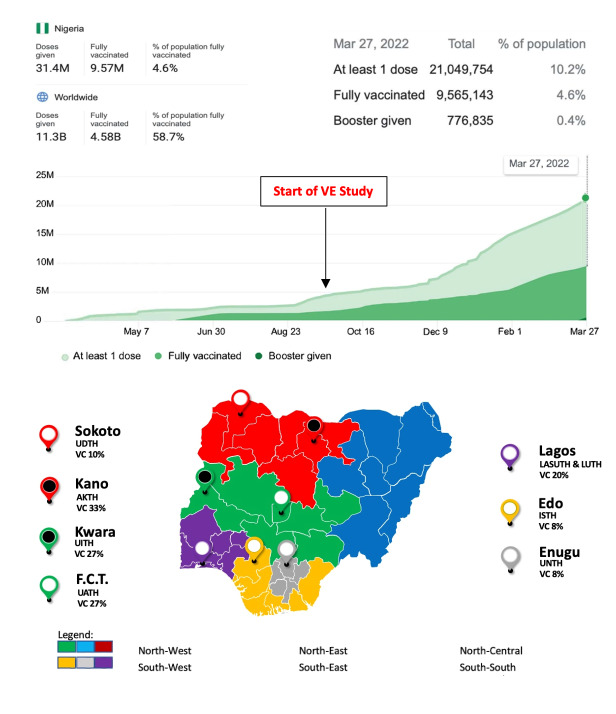
Progression of COVID-19 vaccination in Nigeria and distribution of selected health facilities across the geopolitical zones. VC – vaccination coverage among the eligible population in the selected (with at least one dose of the vaccine) as of 5 April 2022, UDTH – Usmanu Danfodio Teaching Hospital, LASUTH – Lagos State University Teaching Hospital, AKTH – Aminu Kano Teaching Hospital, LUTH – Lagos University Teaching Hospital, UITH – University of Ilorin Teaching Hospital, ISTH – Irrua Specialist Teaching Hospital, UATH – University of Abuja Teaching Hospital, UNTH – University of Nigeria Teaching Hospital.

A detailed exposition of the various COVID-19 VE strategies and research being conducted globally has been recently published by the WHO [10]. The TNCC was used in 142 out of the 1161 listed studies. So far, 6 studies have been conducted in sub-Saharan Africa, a third of them adopted the TNCC study design. For our study in Nigeria, recruitment of study participants began in September 2021 across 5 geopolitical zones, with a planned spread across all 6 geopolitical zones to ensure national representativeness (**Figure 2**).

## IMPLEMENTATION REALITIES IN THE NIGERIAN CONTEXT: LESSONS FOR SCIENCE

### Low COVID-19 mortality and morbidity

Nigeria has experienced relatively low morbidity and mortality of COVID-19, compared with the majority of countries in the world (particularly the global north) [10]. As of April 2022, Nigeria had reported about 255 500 cases of the disease, with a test positivity rate of about 5% and a mortality rate of less than 1.3% [11]. However, SARS-COV-2 seroprevalence data indicate significantly higher exposure and transmission in parts of Nigeria in the first eight months of the COVID-19 epidemic in Nigeria with seroprevalence ranging from 9.3% (95% Cl = 7.0-11.5) in Gombe in the northeast to 25.2% (95% CI = 21.8-28.6) in Enugu in the southeast. SARS-CoV-2 antibody prevalence suggests infections exceeded reported cases by 134:1 and 1211:1 in Gombe and Nasarawa, respectively [12]. This is in tandem with similar reports from across the world, i.e., COVID-19 cases numbers are underreported [13].

These realities have an impact on the implementation of VE studies in the country. Low testing rates and the consequent underreporting of cases reduce the pool of individuals who may be identified as eligible to participate in the study when using a TNCC study design. The ensuing challenge is that the minimum sample size may not be reached within the set timeline for the study, and lower sample size will negatively impact the statistical power of the study and/or precision of the measure of effect.

### Low vaccination coverage

According to the initial vaccination plan, 40% of Nigerians would have been vaccinated by the end of 2021 [14]. As of April 2022, however, 17.3% and 11.5% of the eligible population have received at least one and two doses respectively of the COVID-19 vaccine [15]. Ideally, VE studies should be implemented soon as vaccination is rolled out. Despite obtaining ethical approval to begin the study in February 2021, study implementation (participant recruitment) did not begin until September 2021 due to the limited availability of vaccines in the country and the consequent low vaccination coverage. Poor vaccination coverage reduces the feasibility of conducting VE evaluations of infection and transmission and may be overcome by the use of adaptive designs in evaluating VE in settings [9].

### Mixing of vaccine brands and introduction of vaccine booster doses

In the face of an evolving understanding of the immunogenicity of COVID-19 vaccines, Nigeria introduced booster doses in December 2021 with a schedule that inevitably implies the mixing of vaccine brands. This inadvertently limits our ability to infer attribution of VE to a single brand but rather to the omnibus of available vaccines. More detailed analytical designs such as pre-and-post immunological studies would otherwise be required to isolate individual benefits of vaccine brands.

### Building bridges across silos in the conduct of COVID-19 vaccine effectiveness studies

Disparate groups are conducting COVID-19 VE and safety studies in the country. Considering the resource-intensive nature of these studies, this approach may result in duplicated efforts and inefficiency. Under good leadership and coordination, building bridges to link silos in this research subject could lead to improved effectiveness and efficiency in the conduct of COVID-19 VE research in the country.

Consequently, the NCDC is supporting the NCRC in mapping COVID-19 Vaccine Safety and Effectiveness studies being planned or ongoing in the country, to form a national research coordinating team for COVID-19 VE studies in Nigeria. A consultative meeting was held in the latter half of 2021. The meeting had in attendance representatives from key governmental agencies, researchers from more than 20 institutions, and potential local and international funders. Over the next few months, the national research coordinating team plans to facilitate the harmonization of indigenous study protocols, develop a master protocol for research conduct, and actively seek opportunities to fund nationally representative research.

## CONCLUSION

Studying the effectiveness of COVID-19 vaccination in Nigeria presents an essential, but substantial and complex research undertaking. The low COVID-19 hospitalisation and mortality rates, despite the low vaccine coverage rate and resource limitations in the country, raise questions on the value of assessing VE in this setting. However, we find value in assessing VE in Nigeria, particularly in this scenario where COVID-19 vaccine trials were not conducted in-country. The results will contribute to demand creation, and further guide policy and programming around COVID-19 in Nigeria, particularly in addressing the increasing threat of vaccine hesitancy to vaccination uptake. Thus, we recommend the development of unified/master study protocols based on the realities in the continent and the implementation of research priorities in a country with limited resources.

Several studies have shown the effectiveness of COVID-19 vaccines in reducing morbidity and mortality from the disease [16,17]. Routinely reported case data from COVID-19 surveillance in Nigeria also suggests this. Scientific evidence has also demonstrated the cost-saving and cost-effectiveness of COVID-19 vaccination compared with no vaccination across diverse contextual settings [18-20]. Plagued with recurrent outbreaks of infectious diseases and an increasing burden of non-communicable diseases, Nigeria grapples with competing health research needs that require significant resource allocation. Thus, implementing low-cost routine passive surveillance activities that assess the effectiveness of COVID-19 vaccination beyond one-off analytical investigations may be a better option. Finally, we call on the NCDC to use its convening role to create coalitions among currently disparate groups conducting COVID-19 vaccine safety and effectiveness research in the country, for broader national relevance and efficient use of scarce resources.
